# Supplementary material for: Current Induced Resistive State in Fe(Se,Te) Superconducting Nanostrips
Source: Sci Rep. 2017 Jun 23;7:4115. doi: 10.1038/s41598-017-04425-x (PMC5482902; doi:10.1038/s41598-017-04425-x)
Supplement: Supplementary file 1 — Supplementary Information [file 41598_2017_4425_MOESM1_ESM.pdf]

Current Induced Resistive State in Fe(Se,Te) Superconducting Nanostrips

Ciro Nappi[1,*] , Carlo Camerlingo[1], Emanuele Enrico[2], Emilio Bellingeri[3], Valeria Braccini[3], Carlo Ferdeghini[3], and Ettore Sarnelli[1,+]

[1]CNR-SPIN, Sede Secondaria di Napoli, I-80078 Pozzuoli, Napoli (NA), Italy

[2]INRIM, Istituto Nazionale di Ricerca Metrologica, I-10135 Torino, Italy

[3]CNR-SPIN, Genova, Corso Perrone 24, I-16152 Genova, Italy

*ciro.nappi@spin.cnr.it

+ettore.sarnelli@spin.cnr.it

Supplementary Information

S1. Relation between H_{c1} and j_c in a rectangular cross section superconducting nanostrip

In large superconducting samples, the current density associated to the field H_{c1} is[26]

$$j_c = \frac{H_{c1}}{\lambda} \tag{s1}$$

so that, if one assume equation (s1), j_c is fundamentally determined just by λ and ξ and independent of the pinning microstructure or the geometry of the sample. In our rectangular cross section nanostrips, where $d,\ w \lesssim \lambda$, the use of the equation (s1) for evaluating the critical current density, is not allowed, in principle; the critical current density is sample dependent and equation (s1) is substituted by more *ad hoc* relations. In order to establish a relation between H and j (and between H_c and j_c) in our nanostrips we have used a simple model in which a rectangular cross section superconducting nanostrips, with $w,d \lesssim \lambda$, bearing a uniform current density, is approximated by a series of $n = w/d$ parallel wires with a diameter equal to the film thickness d, each carrying the same current density $j = 4I_b/(n\pi d^2)$. The latter assumption is a direct consequence of the hypothesis $w,d \lesssim \lambda$. The Ampère law gives for the magnetic induction B at a distance x from the centre of a wire carrying a current I, the value $B = \mu_0 I/2\pi x$. Then at the edge (left or right) of a strip made by n parallel wires we can write the magnetic field $H = B/\mu_0$ as a sum of the n magnetic fields generated by each wire at the edge:

$$H = \frac{I}{\pi d}\left(1+\frac{1}{3}+\frac{1}{5}+\cdots\frac{1}{2n-1}\right) = \frac{I_b}{n\pi d}\left(1+\frac{1}{3}+\frac{1}{5}+\cdots\frac{1}{2n-1}\right) = \frac{jd\alpha}{4} \tag{s2}$$

In this way the relation between magnetic field and current density at the left and right edge of the nanostrip may be written as $H = \alpha d\, j/4$ where, for sample A (n=5), $\alpha_A = 1.78$ while for sample B (n=8), $\alpha_B = 2.02$. These numbers are, of course, model dependent and in particular it can be shown that the 'wire' model tends to underestimate the edge magnetic field of a rectangular cross section

strip. Note that for the presence of the superconducting banks overlooking the strip-line the α values found above should be doubled for a net focussing effect due to the image currents of the bias current in the banks, i.e. $\alpha_A = 1.78 \times 2$ and $\alpha_B = 2.02 \times 2$.

The effect of the presence of corners can also be evaluated. Clem and Berggren [30] calculate the amplification factor of the magnetic field due to a 90° corner in a situation like the one shown in Fig. 1b as a factor $\gamma = 2/3(w/\pi\,\xi)^{1/3}$ providing for the two samples the values γ_A=2.86 and γ_B=3.85. Thus the relation between the magnetic field H and the current density j at the surface of the sample in the situation of our experiments can be approximated as

$$j_c = \frac{4H_{c1}}{\alpha\gamma d} \qquad \text{(s3)}$$

which is equation (2) of the main part.

S2. On the creep velocity formula equation (5)

U is assumed to be the difference in Gibbs energy when a flux-line intersects a defect and when it is out of the defect. The motion of a magnetic flux line in the presence of thermal fluctuations is determined by the escape rate from the well of depth *U*. This latter is given by $\omega = \omega_0\, exp(-U/k_BT)$ where ω_0 is the attempt frequency. In the presence of a current making a work W on the magnetic flux line, there are two hopping rates[33]: a forward hopping rate, $\omega_f = \omega_0\, exp(-(U-W)/k_BT)$, and a backward hopping rate, $\omega_b = \omega_0\, exp(-(U+W)/k_BT)$; the difference between the two rates determines the average creep flow velocity $v = (\omega_f - \omega_b)\delta$:

$$v = (\omega_f - \omega_b)\delta = 2v_0 \exp\left(-\frac{U}{k_BT}\right)\sinh\left(\frac{W}{k_BT}\right) \qquad \text{(s4)}$$

where $v_0 = \omega_0\delta$ and δ is the effective pinning potential range. When the condition $W >> k_BT$ holds, that is when forward hopping prevails on the backward hopping, since $\sinh(x) \approx \exp(x)/2$,

$$v = v_0 \exp\left(-\frac{U}{k_B T}\right)\exp\left(\frac{W}{k_B T}\right).^{33} \qquad \text{(s5)}$$

The highest temperature used in our experiments is $T = 13$K, which corresponds to an energy $k_B T$ ~1meV. The least current intensity we use in our analysis is I_b = 10mA (sample A), corresponding to a minimum current density of 2×10^8 A/m^2. The minimum work is then estimated to be $j_b d\phi_0\delta$ ~ 5 meV, implying for the maximum difference between (s4) and (s5) the value exp(5)/2 - sinh(5) ≈ 0.003. Analogous considerations apply to sample B. Then in our measurements $W >> k_B$T holds all the time. This largely justify the use of equation (s5), i.e. equation (5) of the main part, instead of (s4).

References cited in Supplementary Information (keeping their original numbers used in the main part)

26. Tinkham M., Introduction to Superconductivity (Dover Publication, 1996)

30. Clem J. R. and Berggren K. K. Geometry-dependent critical currents in superconducting nanocircuits *Phys. Rev. B* **84** 174510 (2011)

33. Dew-Hughes D. Model for flux creep in high Tc superconductors *Cryogenics* **28** 674 (1988)
